# Development and Validation of an Interpretable Machine Learning Model for Staging Helicobacter pylori–Initiated Intestinal-Type Gastric Cancer in the Correa Cascade: Cross-Sectional Study

**DOI:** 10.2196/94837

**Published:** 2026-08-07

**Authors:** Jiawei Tang, Huijin Chen, Wenwen Zhang, Alfred Chin Yen Tay, Barry J Marshall, Cong Ma, Liang Wang

**Affiliations:** 1 The Marshall Centre for Interventions in Infectious Disease, The University of Western Australia, Perth, Australia; 2 Department of Laboratory Medicine, Shengli Oilfield Central Hospital, Dongyin, Shandong, China; 3 Department of Clinical Medicine, School of the 1st Clinical Medicine, Xuzhou Medical University, Xuzhou, Jiangsu, China; 4 Marshall Research Centre for Medical Microbial Biotechnology, Department of Life Sciences, Faculty of Science, The Hong Kong Polytechnic University (PolyU), Hongkong, China (Hong Kong); 5 Department of Laboratory Medicine, Guangdong Provincial People's Hospital, Guangdong Academy of Medical Sciences, Southern Medical University, No. 106, Zhongshan 2nd Road, Yuexiu District, Guangzhou, Guangdong, 510080, China, 86 13921750542; 6 School of Medicine, South China University of Technology, Guangzhou, Guangdong, China; 7 Department of Intelligent Medical Engineering, School of Medical Informatics and Engineering, Xuzhou Medical University, Xuzhou, Jiangsu, China; 8 School of Molecular Sciences, School of Biomedical Sciences, The University of Western Australia, Perth, Australia

**Keywords:** intestinal-type gastric cancer, routine laboratory indicators, machine learning, hematological parameters, Correa cascade, *Helicobacter pylori*

## Abstract

**Background:**

Gastric cancer (GC) is one of the most common malignant tumors worldwide, with *Helicobacter pylori*–associated intestinal-type gastric cancer (IGC) being the most prevalent subtype, accounting for approximately 85% of cases. Because most patients are diagnosed at intermediate or advanced stages, early screening and accurate stage stratification of IGC progression remain major clinical challenges.

**Objective:**

This study aimed to develop an interpretable machine learning (ML) model that leverages routine laboratory indicators to perform stage-specific diagnosis for patients across different stages of IGC.

**Methods:**

Data from 2180 patients with known *H pylori* infection status were collected at 2 centers and included healthy controls (HCs), nonatrophic gastritis, atrophic gastritis, intestinal metaplasia, and GC. After excluding cases with severe (>25%) missing data, 1784 patients were included for model development and validation. Data imputation and feature selection were performed, and synthetic minority oversampling technique (SMOTE) augmentation was applied to the internal training dataset to improve the diagnostic performance of the model. Six ML algorithms were developed. Model performance and clinical decision-making utility were evaluated using multiple metrics and approaches, while Shapley Additive Explanations (SHAP)–based interpretability was used to identify key indicators and provide threshold reference values for them. Finally, a web-based tool was developed based on the Streamlit platform.

**Results:**

Through feature selection, 27 features were ultimately retained for final model construction. Among the 6 algorithms, CatBoost (categorical boosting) demonstrated the best performance, achieving an internal validation accuracy of 80.91%, sensitivity of 78.57%, and specificity of 95.27%. In the Guangdong Provincial People’s Hospital (GDPH) and Shengli Oilfield Central Hospital (SOCH) external validation cohorts, CatBoost maintained robust performance, with accuracies of 79.96% and 83.37%, sensitivities of 76.82% and 84.91%, specificities of 93.14% and 95.73%, and area under the curves (AUCs) of 0.94 and 0.97, respectively. Confusion matrix analysis showed that the model was particularly reliable in identifying extreme disease states, including HCs and GC, whereas misclassifications mainly occurred between adjacent intermediate pathological stages. Calibration curves and Brier scores indicated good agreement between predicted and observed outcomes. Decision curve analysis (DCA) further confirmed the clinical net benefit across relevant threshold ranges. SHAP-based interpretability analysis identified monocyte count (MONO%), albumin/globulin ratio (A/G), basophil percentage (BASO%), platelet distribution width (PDW), total bilirubin (DBIL), neutrophil count (NEUT#), age, lymphocyte count (LYMPH#), creatinine (CREA), and aspartate aminotransferase (AST) as important contributors, reflecting inflammatory, hematological, nutritional, and metabolic changes during IGC progression. Based on these features, a lightweight predictive model was developed and deployed as a web-based application to facilitate translational and practical applications.

**Conclusions:**

This study developed an interpretable ML model based on routine laboratory data for stage-specific prediction of *H pylori*–associated IGC progression, with promising applicability as an auxiliary diagnostic tool.

## Introduction

According to the latest GLOBOCAN estimates from the International Agency for Research on Cancer (IARC), approximately 968,000 new gastric cancer (GC) cases and 660,000 related deaths occurred worldwide in 2022. Both the incidence and mortality of GC rank fifth globally [1]. East Asia, Eastern Europe, and South America are high-incidence regions for GC, with the disease burden particularly pronounced in East Asian countries. Data from the National Cancer Center of China (NCC) indicate that approximately 358,700 new GC cases and about 260,400 deaths occurred in China in 2022 [2]. From a histopathological perspective, Danish pathologist Pekka Antero Laurén classified GC into intestinal-type gastric cancer (IGC) and diffuse-type GC in 1965 [3]. *Helicobacter pylori* infection is considered the initiating and essential factor for the development of IGC, as it colonizes the gastric mucosa, triggering active gastritis [4], and gradually driving disease progression to atrophic gastritis (AG), intestinal metaplasia (IM), dysplasia, and ultimately IGC [5]. Due to the insidious clinical symptoms of early GC, most patients are diagnosed at an advanced stage, missing the optimal opportunity for curative surgery. Even among those who undergo surgical treatment, approximately two-thirds remain at risk of recurrence or metastasis. Large-scale cohort studies suggest that the optimal window for GC intervention lies before the onset of IM [6]. Therefore, early detection and intervention of IGC is crucial for improving patient prognosis [7].

Endoscopic examination is the cornerstone of GC diagnosis, but conventional white-light gastroscopy heavily depends on the operator’s experience, making it difficult to promptly identify gastric mucosal atrophy and IM [8]. Although a combined biopsy is the gold standard for GC diagnosis [9], its invasiveness and the expensive medical resources limit its use in widespread screening. Multiomics analyses based on the microbiome and metabolome can characterize differential microbial communities and metabolites during disease progression. These alterations may provide potential biomarkers for the early diagnosis of IGC [10]. However, similar to endoscopy, these methods are costly and time-consuming, making them unsuitable for large-scale, on-site screening. AI-based pathological image analysis can assist clinicians in diagnosis and has been proven capable of accurately detecting GC [11]. For example, Song et al [12] trained a deep learning model using 2123 pixel-level stained whole-slide images, achieving nearly 100% sensitivity and 80.6% specificity in real-world test data. However, high-quality AI diagnostic models for pathology rely on massive manual annotations, requiring substantial labor, time, and computing power, which hinders the iterative development of such technologies. Hematologic indices, owing to their clinical accessibility and quantifiability, have increasingly been used to explore noninvasive biomarkers of inflammation and cancer. For example, red cell distribution width (RDW) has been associated with atherosclerosis, inflammatory bowel disease, and multiple cancers [13]. Mean corpuscular volume (MCV) has been used to assess the prognosis of esophageal cancer [14], whereas hematocrit (HCT) performs comparably to hemoglobin (Hb) in predicting mortality risk in triple-negative breast cancer [15]. Because immune-inflammatory responses can promote angiogenesis and stimulate tumor cell proliferation, invasion, and metastasis, they are directly related to tumor initiation and progression [16]. Current hematologic studies on the early diagnosis of GC have mainly focused on the diagnostic and prognostic value of inflammatory markers [17,18]. Studies show that peripheral blood inflammatory indices are associated with overall survival in patients with locally advanced GC. Platelet-to-lymphocyte ratio (PLR), neutrophil-to-lymphocyte ratio (NLR), and lymphocyte counts often indicate a poorer prognosis [19]. Meanwhile, inflammatory markers such as interleukin-6 (IL-6), C-reactive protein (CRP), and procalcitonin (PCT) are markedly elevated in GC and can provide auxiliary evidence for diagnosis and staging [20]. In addition, several routine blood count parameters have been reported: RDW is increased, while PDW is decreased in patients with GC [13]. A long-term follow-up study has also found that an elevated white blood cell (WBC) count is associated with an increased risk of GC, particularly among individuals infected with *H pylori* [21]. However, most existing studies relied on conventional statistical methods and analyzed only a limited number of hematologic indices, making it difficult to capture complex interindicator associations and latent patterns. Therefore, more advanced computational approaches are urgently needed to fully realize the potential of hematologic indices in the early identification and stratified diagnosis of GC.

Unlike simple statistical analyses based on a single or a few indicators, machine learning (ML) techniques, with their ability to efficiently model multidimensional features and perform pattern recognition, can be deeply integrated with hematological parameters, greatly expanding their clinical diagnostic value [22,23]. For example, Yang et al [1] analyzed 57 hematological indicators from patients with nasopharyngeal, esophageal, and lung cancers undergoing immunosuppressive therapy, and the ML model achieved a sensitivity of 85% and a specificity of 79%; Zhang et al [24] integrated 58 hematological and biochemical indicators from 2951 samples to construct an ML model, obtaining a sensitivity of 85.44% and a specificity of 83.82% in the cross-validation cohort. However, the feasibility of these indicators for staging-specific diagnosis of IGC within the framework of the Correa cascade has not yet been investigated. This retrospective multicenter study aimed to develop and validate an interpretable ML-based diagnostic framework. It was designed for stage-specific diagnosis of *H pylori*–associated IGC progression along the Correa cascade using routine laboratory indicators. Specifically, we used demographic information, complete blood count parameters, and various biochemical indicators from 2 independent centers. We sought to compare imputation methods and data augmentation techniques in terms of data quality, optimize and evaluate multiple ML algorithms, identify the most suitable diagnostic model, and interpret the contribution of key clinical indicators to model decision-making. Subsequently, we aimed to develop a user-friendly online diagnostic platform for the preliminary screening of IGC at different stages of the Correa cascade. This model is expected to assist in the early screening of different stages of IGC progression, especially in resource-limited primary health care settings.

## Methods

### Study Population

This retrospective study aimed to construct and validate a predictive model for classifying disease stages along the *H pylori*–associated Correa cascade. Data were obtained from the laboratory information systems (LIS) of 2 independent, large-scale, grade 3A hospitals, Guangdong Provincial People’s Hospital (GDPH) and Shengli Oilfield Central Hospital (SOCH), between January 2022 and May 2025. The collected data included demographic information, complete blood counts, and serum biochemical markers. *H pylori* infection status was confirmed through gastric mucosal histological examination. Participants were classified into 5 groups, including healthy control (HC), nonatrophic gastritis (NAG), AG, IM, and GC. All diagnoses were independently determined by 2 experienced pathologists according to pathological standards. HCs were recruited from individuals undergoing routine health examinations who had normal upper gastrointestinal endoscopic findings and negative *H pylori* test results. The same exclusion criteria were applied to both HCs and patients across all disease stages, including a history of gastric resection or other gastrointestinal surgeries, coexisting malignancies, a history of organ transplantation, or severe heart, lung, liver, kidney, or hematologic diseases.

### Data Preprocessing

Features with more than 25% missing values were excluded. Missing values in the remaining features were imputed using 6 methods, including mean imputation, median imputation, k-nearest neighbor (KNN) imputation, linear interpolation, multiple imputation, and regression imputation. The imputation results were compared using box plots and histograms to evaluate the impact of different imputation methods on the data distribution and statistical characteristics. Furthermore, considering the potential influence of multicollinearity between features on prediction accuracy, features with a high correlation in the Spearman correlation analysis were excluded if they had a lower correlation with the target variable. Ultimately, the following features were retained, including gender, age, aspartate aminotransferase (AST), albumin (ALB), albumin/globulin ratio (A/G), total bilirubin (DBIL), alkaline phosphatase (AKP), gamma-glutamyl transferase (GGT), glucose (GLU), urea (UREA), creatinine (CREA), uric acid (UA), total cholesterol (TC), triglycerides (TG), high-density lipoprotein cholesterol (HDL), monocyte percentage (MONO%), basophil percentage (BASO%), neutrophil count (NEUT#), lymphocyte count (LYMPH#), monocyte count (MONO#), eosinophil count (EO#), red blood cell (RBC) count, mean corpuscular hemoglobin (MCH), mean corpuscular hemoglobin concentration (MCHC), platelet count (PLT), mean platelet volume (MPV), and platelet distribution width (PDW). To address the issue of class imbalance, we used the synthetic minority over-sampling technique (SMOTE) to generate synthetic samples. SMOTE was applied only to the internal training set, whereas the internal validation set and the 2 external validation cohorts were kept unaugmented. By reducing the bias toward the majority class during model training, this approach enables more stable learning of minority-class patterns and effectively improves the model’s sensitivity to rare but clinically meaningful cases [25,26]. In subsequent analyses, we compared and evaluated the effectiveness of this method.

### Model Selection and Performance Evaluation

This study was conducted using Python 3.9.7, along with the *scikit-learn*, *catboost*, *lightgbm*, and *xgboost* packages for training ML models. The 6 ML algorithms used included adaptive boosting (AdaBoost, *AdaBoostClassifier*), categorical boosting (CatBoost, *CatBoostClassifier*), decision tree (DT, *DecisionTreeClassifier*), light gradient boosting machine (LGBM, *LGBMClassifier*), random forest (RF, *RandomForestClassifier*), and eXtreme gradient boosting (XGBoost, *XGBClassifier*). The training cohort from 2 hospitals was divided into 2 subsets, with 80% allocated as the internal training set and 20% as the internal validation set. Before formal training, all models underwent hyperparameter tuning using *GridSearchCV* to fit all combinations of model parameters (Multimedia Appendix 1), followed by 5-fold cross-validation (CV) for optimal hyperparameter selection. Each model was then trained using the optimal parameter combination. Performance was evaluated via accuracy, macro precision, specificity, macro recall (sensitivity), and macro *F*_1_-score. Receiver operating characteristic (ROC) curve and confusion matrix were also generated to evaluate discrimination and class-specific performance. All models followed the same construction and optimization process to assess the quality of the new data generated by SMOTE preprocessing, thus validating the feasibility of this approach. The best diagnostic model, selected after comparison of all models, was further evaluated using a calibration curve to determine whether the model’s predicted probabilities align with the actual outcomes. Additionally, the model’s potential clinical value was measured using decision curve analysis (DCA). Finally, an external validation cohort was simultaneously collected from both hospitals, with inclusion and exclusion criteria that matched those of the training cohort. The external validation data were entered into the best diagnostic model, and the same evaluation metrics were used to assess the model’s performance on unseen data.

### Interpretability Analysis

To effectively interpret the model’s decision results, the study uses Shapley Additive Explanations (SHAP) for interpretability analysis. SHAP values for each feature are calculated using *TreeExplainer* from the SHAP library. Feature importance is ranked using the *summary_plot*, which provides a visual representation of the overall contribution of each feature to the model’s predictions. The *dependence_plot* function further analyzes the impact of individual features on model predictions, examining the trend of feature changes across different categories and their relationship with the SHAP values. The ridge plot displays the distribution of each feature across different groups, visually highlighting the differences between categories.

### Web Page Deployment Tool Based on Streamlit Framework

To facilitate the clinical application of the developed method, we integrate the best model, constructed from the top 10 features identified by SHAP, into a tool developed using the *Streamlit* framework. A web page deployment tool is created for predictive analysis at different stages of the Correa model. Users can input the corresponding feature values into the model and click the “Predict” button. The tool will return the prediction result and save it as the most recent prediction record.

### Ethical Considerations

This study was approved by the ethics review committee of Guangdong Provincial People’s Hospital (KY2025-1060-01). As this was a retrospective study, the requirement for informed consent was waived. To ensure privacy protection, all data were deidentified using participant codes, and no identifiable information was disclosed.

## Results

### Patient Characteristics

**Figure 1.**
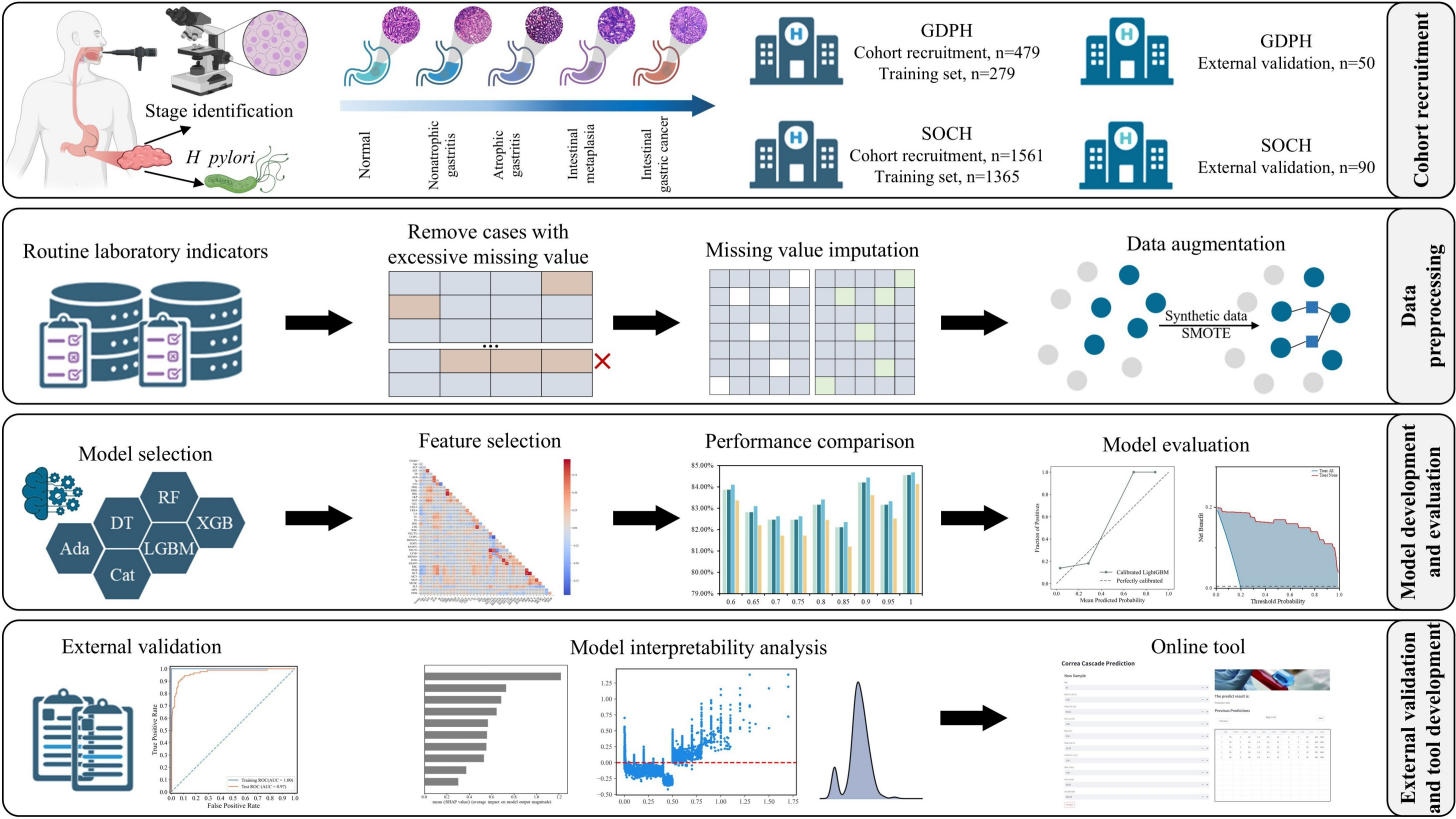
The overall framework of the study. The study primarily includes cohort recruitment, data preprocessing, model construction, and evaluation, as well as external validation and tool development. Ada: adaptive boosting; Cat: categorical boosting; DT: decision tree; GDPH: Guangdong Provincial People’s Hospital; LGBM: light gradient boosting machine; RF: random forest; SOCH: Shengli Oilfield Central Hospital; XGB: eXtreme gradient boosting.

This retrospective analysis included 2180 patients at different stages of *H pylori*–associated GC along the Correa cascade, who were enrolled in the cohort for predictive model construction. During the study, GDPH and SOCH excluded 200 and 196 patients with extensive missing data, respectively. Ultimately, data from 1784 patients were used for model training and external validation. The training cohort included 1644 patients, consisting of 53 HC, 723 NAG, 181 AG, 606 IM, and 81 GC (Table 1). Baseline information after SMOTE augmentation is shown in Multimedia Appendix 2. At GDPH and SOCH, data from 50 and 90 patients were collected, respectively, as external validation cohorts. The overall framework of the study is illustrated in Figure 1, and the detailed study design workflow is provided in Multimedia Appendix 3.

**Table 1.** Baseline characteristics of training sets in Guangdong Provincial People’s Hospital (GDPH) and Shengli Oilfield Central Hospital (SOCH) cohorts.

Characteristics	Training set (N=1644)					*P* value^a^
	HC (n=53)	NAG (n=723)	AG (n=181)	IM (n=606)	GC (n=81)	
Male, n (%)	19 (35.80)	431 (59.61)	104 (57.46)	404 (66.67)	51 (62.90)	<.001
Age (y)	57.66 (12.23)^b^	53.00 (47.00‐63.00)^c^	59.28 (11.19)^b^	57.50 (52.00‐68.00)^c^	62.54 (10.66)^b^	<.001
AST^d^	21.08 (4.40)^b^	20.00 (17.00‐24.00)^c^	21.00 (17.00‐25.00)^c^	21.00 (17.00‐25.00)^c^	19.00 (16.00‐23.00)^c^	.13
ALB^e^	42.45 (4.26)^b^	41.88 (39.20‐44.70)^c^	40.69 (38.15‐43.54)^c^	41.90 (39.50‐44.30)^c^	39.56 (5.33)^b^	<.001
A/G^f^	1.60 (1.50‐1.75)^c^	1.60 (1.40‐1.80)^c^	1.50 (1.30‐1.70)^c^	1.60 (1.50‐1.80)^c^	1.45 (0.28)^b^	<.001
DBIL^g^	3.10 (2.35‐4.25)^c^	2.50 (1.90‐3.30)^c^	2.40 (1.88‐3.20)^c^	2.50 (2.00‐3.50)^c^	2.40 (1.70‐3.50)^c^	.003
AKP^h^	74.00 (60.50‐87.00)^c^	69.00 (56.00‐84.00)^c^	71.00 (59.00‐88.00)^c^	70.00 (59.00‐84.00)^c^	72.00 (59.00‐90.02)^c^	.23
GGT^i^	21.00 (15.00‐28.00)^c^	23.00 (16.00‐39.00)^c^	24.00 (15.00‐39.00)^c^	22.00 (16.00‐38.00)^c^	19.00 (15.00‐31.00)^c^	.15
GLU^j^	5.11 (4.61‐5.70)^c^	5.11 (4.61‐5.78)^c^	5.14 (4.62‐5.93)^c^	5.12 (4.62‐5.88)^c^	5.14 (4.71‐6.05)^c^	.73
UREA^k^	5.30 (3.94‐6.15)^c^	4.96 (4.28‐5.83)^c^	5.22 (4.23‐6.35)^c^	5.22 (4.43‐6.30)^c^	5.69 (4.61‐6.60)^c^	.001
CREA^l^	55.80 (48.80‐67.10)^c^	64.70 (54.30‐76.80)^c^	66.60 (56.71‐82.63)^c^	66.80 (55.60‐76.93)^c^	67.00 (55.73‐78.60)^c^	<.001
UA^m^	309.70 (105.00)^b^	336.80 (266.10‐403.00)^c^	345.00 (288.10‐414.95)^c^	330.10 (270.98‐396.00)^c^	309.82 (94.87)^b^	.004
TC^n^	4.87 (0.99)^b^	4.95 (4.16‐5.69)^c^	5.11 (4.27‐5.73)^c^	4.88 (4.21‐5.62)^c^	4.67 (1.14)^b^	.10
TG^o^	1.22 (0.92‐1.54)^c^	1.33 (0.98‐1.96)^c^	1.36 (0.97‐2.09)^c^	1.36 (0.99‐1.95)^c^	1.26 (0.91‐1.76)^c^	.24
HDL^p^	1.42 (1.12‐1.68)^c^	1.19 (1.00‐1.46)^c^	1.23 (1.04‐1.43)^c^	1.2 (1.02‐1.47)^c^	1.43 (0.48)^b^	.03
MONO%^q^	6.80 (5.50‐8.00)^c^	6.80 (5.00‐8.10)^c^	6.30 (0.11‐7.80)^c^	6.70 (5.50‐8.20)^c^	0.13 (0.07‐7.00)^c^	<.001
BASO%^r^	0.40 (0.20‐0.60)^c^	0.30 (0.10‐0.50)^c^	0.30 (0.01‐0.50)^c^	0.40 (0.20‐0.60)^c^	0.01 (0.005‐0.30)^c^	<.001
NEUT#^s^	3.06 (2.72‐3.63)^c^	3.27 (2.63‐4.35)^c^	3.38 (2.6‐4.07)^c^	3.43 (2.67‐4.35)^c^	4.23 (3.13‐5.57)^c^	<.001
LYMPH#^t^	1.67 (1.49‐2.31)^c^	1.83 (1.48‐2.24)^c^	1.87 (1.52‐2.25)^c^	1.91 (1.46‐2.35)^c^	1.6 (1.33‐2.01)^c^	.007
MONO#^u^	0.36 (0.29‐0.47)^c^	0.42 (0.32‐0.52)^c^	0.44 (0.33‐0.56)^c^	0.42 (0.34‐0.55)^c^	0.48 (0.38‐0.64)^c^	<.001
EO#^v^	0.1 (0.06‐0.14)^c^	0.12 (0.07‐0.20)^c^	0.14 (0.06‐0.24)^c^	0.11 (0.06‐0.20)^c^	0.10 (0.05‐0.19)^c^	.04
RBC^w^	4.46 (0.63)^b^	4.56 (4.20‐4.91)^c^	4.51 (4.13‐4.87)^c^	4.59 (4.25‐4.90)^c^	4.23 (0.73)^b^	.001
MCH^x^	30.10 (29.30‐31.50)^c^	30.10 (29.30‐31.40)^c^	30.60 (29.35‐31.90)^c^	30.70 (29.60‐31.70)^c^	30.50 (28.90‐31.60)^c^	<.001
MCHC^y^	335.77 (12.17)^b^	335.00 (327.00‐343.00)^c^	337.00 (326.00‐344.00)^c^	337.00 (330.00‐344.00)^c^	334.00 (325.00‐342.00)^c^	.002
PLT^z^	229.00 (192.00‐264.00)^c^	228.00 (195.00‐276.00)^c^	220.00 (183.50‐269.00)^c^	216.50 (182.00‐262.25)^c^	237.00 (199.50‐307.50)^c^	.001
MPV^aa^	10.15 (1.09)^b^	10.10 (9.40‐10.60)^c^	10.01 (1.15)^b^	9.90 (9.20‐10.60)^c^	9.70 (0.99)^b^	.03
PDW^ab^	13.60 (11.30‐15.95)^c^	11.90 (10.50‐14.00)^c^	11.90 (10.40‐13.65)^c^	11.70 (10.40‐14.68)^c^	10.90 (9.50‐13.10)^c^	<.001

a *P* value: healthy control (HC) vs nonatrophic gastritis (NAG), atrophic gastritis (AG) vs intestinal metaplasia (IM) vs gastric cancer (GC).

b Mean (SD).

c Median (IQR).

d AST: aspartate aminotransferase.

e ALB: albumin.

f A/G: albumin-to-globulin ratio.

g DBIL: direct bilirubin.

h AKP: alkaline phosphatase.

i GGT: gamma-glutamyl transferase.

j GLU: glucose.

k UREA: urea.

l CREA: creatinine.

m UA: uric acid.

n TC: total cholesterol.

o TG: triglycerides.

p HDL: high-density lipoprotein.

q MONO%: monocyte percentage.

r BASO%: basophil percentage.

s NEUT#: neutrophil count.

t LYMPH#: lymphocyte count.

u MONO#: monocyte count.

v EO#: eosinophil count.

w RBC: red blood cell.

x MCH: mean corpuscular hemoglobin.

y MCHC: mean corpuscular hemoglobin concentration.

z PLT: platelet count.

aa MPV: mean platelet volume.

ab PDW: platelet distribution width.

This study included data from patients with IGC at different stages, collected at GDPH and SOCH. The data were divided into 5 groups: HC, NAG, AG, IM, and GC. The baseline demographic and clinical characteristics of the training cohort are shown in Table 1. Significant differences in gender distribution and age were observed among the 5 groups, with both variables showing statistical significance (*P*<.001). In the disease groups, the proportion of males was higher than in the HC group, particularly in the IM and GC groups. Age progressively increased with disease severity; the highest age values were observed in the GC group and relatively lower values in the NAG group. In contrast, indicators related to metabolism and liver function (AST, AKP, GGT, GLU, TC, and TG) did not show significant differences among the groups (*P*>.05). Several biochemical and hematological parameters exhibited significant differences across disease stages and demonstrated trends associated with different stages of IGC progression. As the disease progressed, ALB levels and the A/G ratio declined overall, reaching the lowest values in the GC group, while renal function indicators (including UREA and CREA) gradually increased, peaking in the GC group. UA levels increased in the early disease stages but decreased in the GC group. HDL levels were significantly lower in the disease groups compared with the HCs. Peripheral blood cell parameters also showed stage-related changes. As disease severity increased, NEUT# and MONO# progressively increased, reaching their highest values in the GC group, whereas LYMPH counts were relatively higher in the precancerous stages but decreased in the GC group. RBC counts were significantly reduced in the GC group, while MCH and MCHC showed mild increasing trends. PLT was relatively elevated in the GC group, while MPV and PDW decreased with disease progression; all these differences were statistically significant. The detailed number of patients in each group and the characteristics of the respective indicators for the 2 external validation cohorts are provided in Multimedia Appendices 4 and 5.

### Data Preprocessing and Validity Assessment

**Figure 2.**
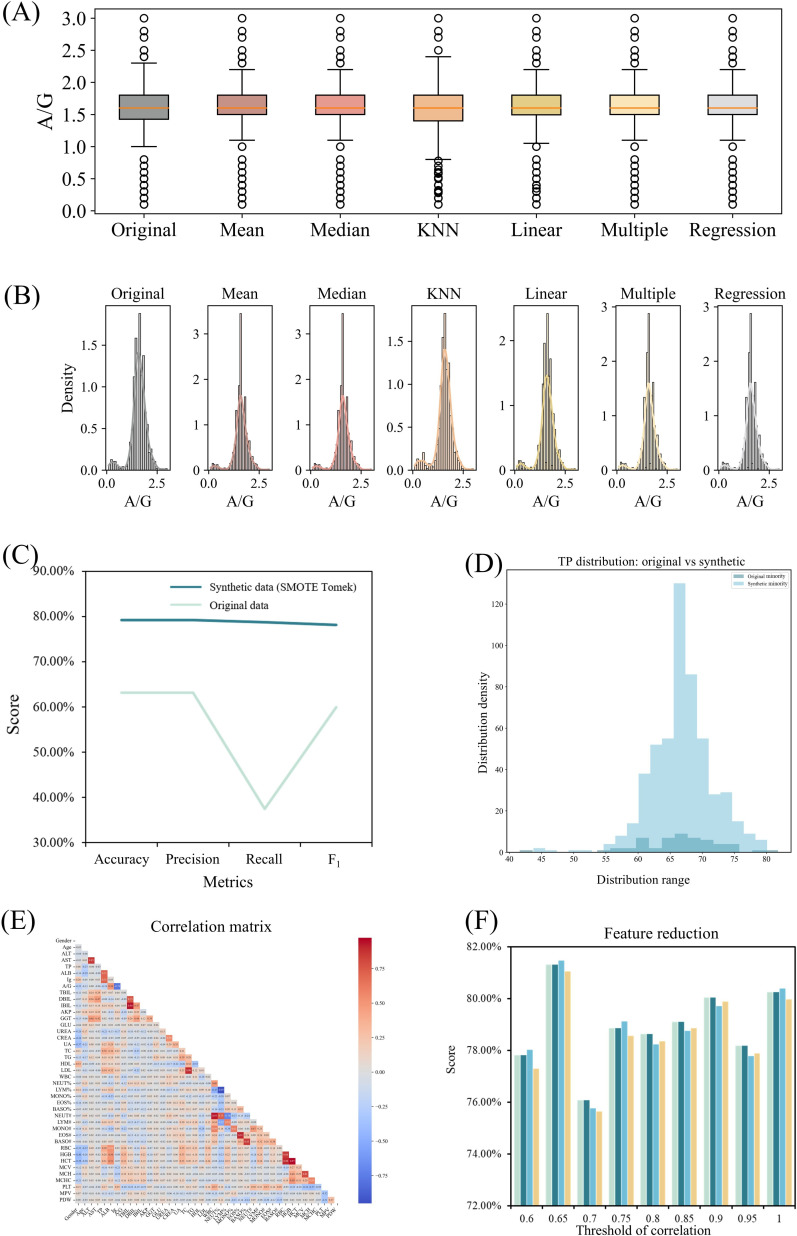
Preprocessing performance evaluation. (A) Box plot comparison of different imputation methods. (B) Density distribution comparison of different imputation methods. (C) Performance comparison before and after SMOTE augmentation. (D) Distribution of SMOTE-augmented data. (E) Feature correlation heatmap. (F) Impact of different feature selection thresholds on model performance. A/G: albumin/globulin ratio; KNN: k-nearest neighbor; SMOTE: synthetic minority oversampling technique; TP: true positive.

To address missing values, 6 imputation methods were applied, and the effects on feature distributions were systematically compared. Using the A/G index as an example, the boxplot results showed similar medians and IQRs across all imputation methods, with no obvious systematic shifts observed (Figure 2A). Further density distribution analyses showed that mean and median imputation smoothed the distributions to some extent, whereas linear, multiple, and regression imputation introduced varying degrees of adjustment to the local density structure (Figure 2B). In contrast, the distribution obtained by KNN imputation was highly consistent with the original data, fully preserving the pronounced bimodal structure and introducing almost no additional artifacts. Therefore, KNN imputation was selected as the optimal method, and the KNN-imputed dataset was used in all subsequent analyses. After completing missing-value imputation, the effects of the original data and SMOTE-augmented data on model performance were further compared. Compared with the nonaugmented data, the model accuracy after SMOTE augmentation increased from approximately 63% to about 80%, indicating that this approach effectively improves the model’s ability to identify minority-class samples (Figure 2C). To assess whether data augmentation introduced potential distributional shifts, a comparative analysis of feature distributions between the original data and the SMOTE-augmented data was conducted (Figure 2D). The results demonstrate that the augmented data remained highly consistent with the original data in overall distribution shape, central tendency, and value range, with no apparent distributional distortion observed. Considering the potential presence of multicollinearity among clinical indicators, correlations among features were further calculated and visualized using a feature correlation heatmap (Figure 2E). Subsequently, the effects of different correlation coefficient thresholds on model performance were evaluated (Figure 2F), and the detailed performance metrics for each threshold are provided in Multimedia Appendix 6. The results indicate that when the correlation coefficient threshold was set to 0.65, the overall model performance reached its optimum, with all evaluation metrics stably maintained at approximately 80%, while retaining 27 features. Therefore, subsequent model construction and analyses were based on this selected feature set.

### Model Comparison and External Validation

The results show that CatBoost performed the best on the internal training data (Table 2), achieving the highest accuracy of 0.9986 (95% CI 0.9978‐0.9993), sensitivity of 0.9997 (95% CI 0.9994‐0.9998), and specificity of 0.9986 (95% CI 0.9977‐0.9993). However, the mean accuracy across 5-fold cross-validation was 0.8087 (SD 0.0024), indicating overfitting and suggesting that the performance may be lower in actual applications. In contrast, AdaBoost performed the worst on the internal training data, with an accuracy of 0.5655 (95% CI 0.5543‐0.5765), which may reflect its limited ability to handle strong correlations among features. In the internal validation data, this overfitting phenomenon was further validated. Although its performance decreased, CatBoost still showed the best performance, with an accuracy of 0.8091 (95% CI 0.7972‐0.8211), specificity of 0.9527 (95% CI 0.9496‐0.9559), and sensitivity of 0.7857 (95% CI 0.7743‐0.7968), demonstrating ideal results. Other models, such as LGBM, also performed well in the internal validation, achieving an accuracy of 0.8067 (95% CI 0.7933‐0.8195), which is similar to that of CatBoost. During the external validation stage, data from GDPH and SOCH were used for initial transportability assessments to evaluate the model’s performance in independent cohorts. Although CatBoost showed some fluctuations in these 2 external validation cohorts, the accuracy for the GDPH cohort was 0.7996 (95% CI 0.6800‐0.9000), sensitivity was 0.7682 (95% CI 0.6866‐0.8518), and specificity was 0.9314 (95% CI 0.8877‐0.9678); for the SOCH cohort, the accuracy was 0.8337 (95% CI 0.7553‐0.9111), sensitivity was 0.8491 (95% CI 0.7812‐0.9082), and specificity was 0.9573 (95% CI 0.9351‐0.9768). The performance was similar to that in the internal validation data, providing preliminary evidence of model transportability for clinical application.

**Table 2.** Comparison of the performance of different ML models on internal training and validation data for diagnosing different stages of the Correa cascade, and the performance of the best model (CatBoost^a^) on 2 external validation cohorts.

Algorithm	Accuracy (95% CI)	Precision (95% CI)	Sensitivity (95% CI)	Specificity (95% CI)	*F*_1_-score (95% CI)	5-Fold CV^b^, mean (SD)
Internal training						
CatBoost	0.9986 (0.9978‐0.9993)	0.9984 (0.9974‐0.9993)	0.9997 (0.9994‐0.9998)	0.9986 (0.9977‐0.9993)	0.9985 (0.9975‐0.9993)	0.8087 (0.0024)
LGBM^c^	0.9986 (0.9977‐0.9995)	0.9986 (0.9977‐0.9994)	0.9984 (0.9974‐0.9993)	0.9997 (0.9994‐0.9999)	0.9985 (0.9975‐0.9994)	0.8065 (0.0083)
RF^d^	0.9986 (0.9977‐0.9993)	0.9986 (0.9977‐0.9994)	0.9984 (0.9972‐0.9993)	0.9997 (0.9994‐0.9998)	0.9985 (0.9975‐0.9994)	0.7980 (0.0000)
XGBoost^e^	0.9985 (0.9975‐0.9993)	0.9984 (0.9974‐0.9993)	0.9983 (0.9971‐0.9992)	0.9996 (0.9994‐0.9998)	0.9984 (0.9973‐0.9993)	0.7854 (0.0074)
DT^f^	0.9891 (0.9865‐0.9915)	0.9895 (0.9871‐0.9918)	0.9884 (0.9858‐0.9911)	0.9972 (0.9966‐0.9978)	0.9942 (0.9924‐0.9959)	0.6356 (0.0123)
AdaBoost^g^	0.5655 (0.5543‐0.5765)	0.5550 (0.5441‐0.5661)	0.5533 (0.5427‐0.5637)	0.8912 (0.8884‐0.8940)	0.5531 (0.5427‐0.5639)	0.4971 (0.0047)
Internal validation						
CatBoost	0.8091 (0.7972‐0.8211)	0.7806 (0.7675‐0.7934)	0.7857 (0.7743‐0.7968)	0.9527 (0.9496‐0.9559)	0.8005 (0.7881‐0.8127)	0.8087 (0.0024**)**
LGBM	0.8067 (0.7933‐0.8195)	0.7824 (0.7698‐0.7950)	0.7860 (0.7743‐0.7978)	0.9524 (0.9489‐0.9557)	0.7837 (0.7712‐0.7956)	0.8065 (0.0083)
RF	0.7930 (0.7796‐0.8063)	0.7615 (0.7466‐0.7772)	0.7679 (0.7564‐0.7806)	0.9485 (0.9451‐0.9518)	0.7538 (0.7404‐0.7664)	0.7980 (0.0000)
XGBoost	0.7857 (0.7727‐0.7989)	0.7572 (0.7432‐0.7705)	0.7638 (0.7516‐0.7752)	0.9469 (0.9435‐0.9503)	0.7587 (0.7457‐0.7711)	0.7854 (0.0074)
DT	0.6344 (0.6183‐0.6491)	0.6098 (0.5945‐0.6251)	0.6163 (0.6017‐0.6307)	0.9091 (0.9050‐0.9128)	0.6112 (0.5966‐0.6264)	0.6356 (0.0123)
AdaBoost	0.4974 (0.4817‐0.5131)	0.4872 (0.4721‐0.5031)	0.4854 (0.4702‐0.5007)	0.8742 (0.8701‐0.8782)	0.4854 (0.4702‐0.5007)	0.4971 (0.0047)
External validation (CatBoost)						
GDPH^h^	0.7996 (0.6800‐0.9000)	0.7996 (0.6800‐0.9000)	0.7682 (0.6866‐0.8518)	0.9314 (0.8877‐0.9678)	0.7889 (0.7003‐0.8703)	N/A^i^
SOCH^j^	0.8337 (0.7553‐0.9111)	0.8337 (0.7553‐0.9111)	0.8491 (0.7812‐0.9082)	0.9573 (0.9351‐0.9768)	0.8439 (0.7713‐0.9063)	N/A

a CatBoost: categorical boosting.

b CV: cross-validation.

c LGBM: light gradient boosting machine.

d RF: random forest.

e XGBoost: extreme gradient boosting.

f DT: decision tree.

g AdaBoost: adaptive boosting.

h GDPH: Guangdong Provincial People’s Hospital.

i Not applicable.

j SOCH: Shengli Oilfield Central Hospital.

**Figure 3.**
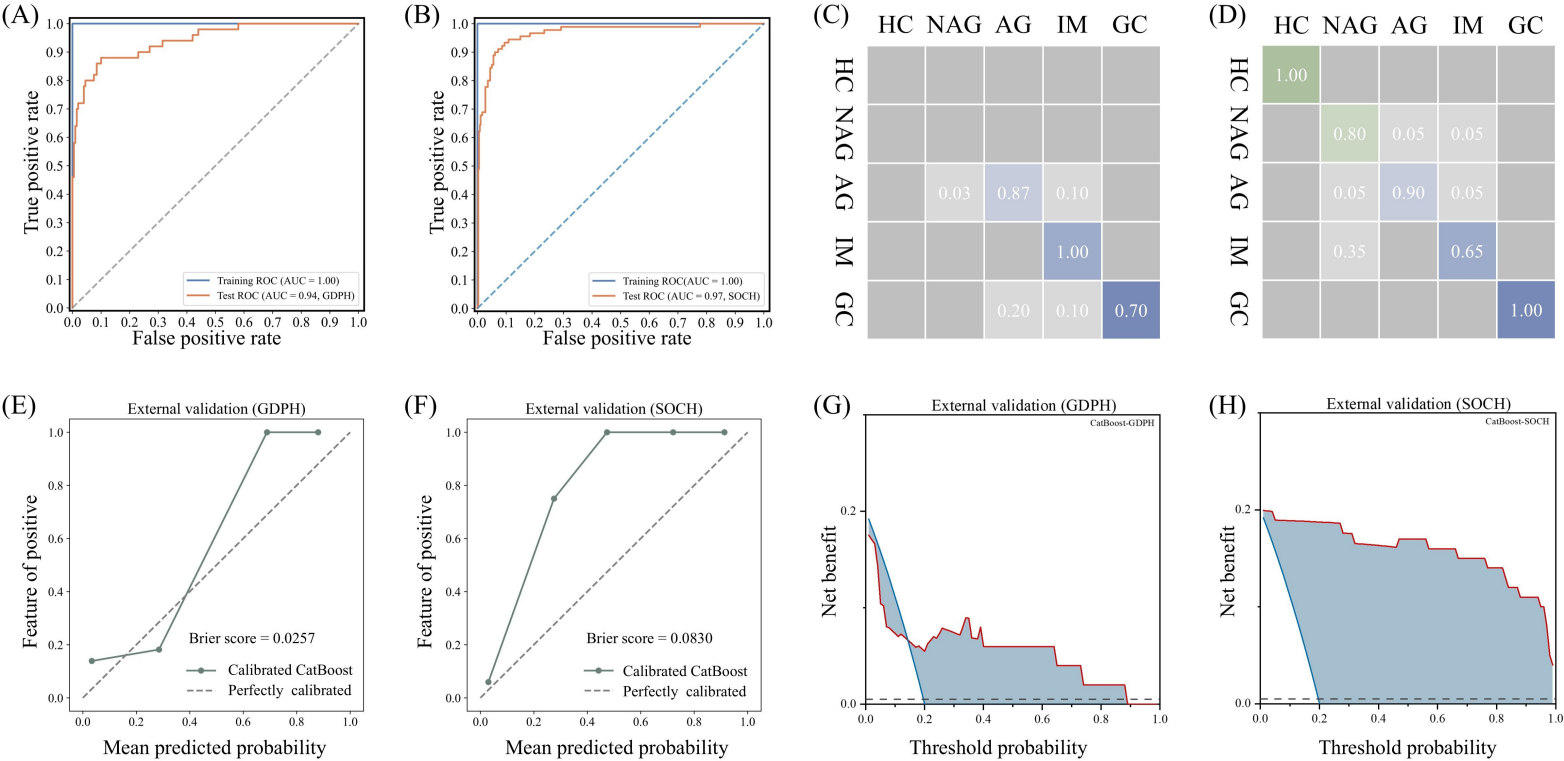
Performance evaluation of CatBoost (categorical boosting) in the external validation cohort. ROC curve in the (A) GDPH and (B) SOCH external validation cohort. Confusion matrix in the (C) GDPH and (D) SOCH external validation cohort. Calibration curve in the (E) GDPH and (F) SOCH external validation cohort. DCA in the (G) GDPH and (H) SOCH external validation cohort. AG: atrophic gastritis; DCA: decision curve analysis; GC: gastric cancer; GDPH: Guangdong Provincial People’s Hospital; HC: healthy control; IM: intestinal metaplasia; NAG: nonatrophic gastritis; ROC: receiver operating characteristic; SOCH: Shengli Oilfield Central Hospital.

In external validation, the CatBoost model demonstrated good discriminative performance across 2 independent centers. In the GDPH external validation cohort, the ROC curve (area under the curve [AUC]) was 0.94 (Figure 3A), while the AUC of the SOCH external validation cohort was 0.97 (Figure 3B), indicating promising discriminative ability of the model at both centers. The confusion matrices illustrated the detailed classification performance across different disease stages. In the GDPH cohort, the correct classification rate for AG was 87%, with approximately 13% of AG samples misclassified into the adjacent IM stage (Figure 3C). The IM category showed the best recognition performance, achieving a 100% correct classification rate. The correct classification rate for GC was 70%, with the remaining 30% mainly misclassified as AG (20%) and IM (10%). In the SOCH cohort, the classification accuracies for HC and GC both reached 100% (Figure 3D). The correct classification rates for NAG and AG were 80% and 90%, respectively, with a small number of samples showing cross-misclassification between NAG and AG. The correct classification rate for IM was 65%, with the remaining 35% misclassified as NAG. Misclassifications in this cohort were concentrated in intermediate pathological stages, suggesting that the model was capable of identifying extreme states (HC and GC). The consistency of probability predictions in the external validation cohorts was assessed using calibration curves and Brier scores. In the GDPH cohort (Figure 3E), the calibration curve closely approximated the ideal reference line, with a Brier score of 0.0257. In the SOCH cohort, the Brier score was 0.0830 (Figure 3F), indicating good agreement between predicted probabilities and observed outcomes. DCA demonstrated that the CatBoost model provided potential clinical utility in both external validation centers. In the GDPH cohort (Figure 3G), when the threshold probability ranged from approximately 0.15 to 0.9, the net benefit of the model consistently exceeded that of the “treat-all” and “treat-none” strategies, with stable positive gains observed in the low-to-moderate threshold range. In the SOCH cohort (Figure 3H), the model maintained a significant net benefit advantage across a wider threshold probability range (approximately 0.02‐0.98), suggesting its potential net benefit advantage. Collectively, these findings suggest that the CatBoost model has the potential to provide effective support for clinical diagnosis.

### Model Interpretation

**Figure 4.**
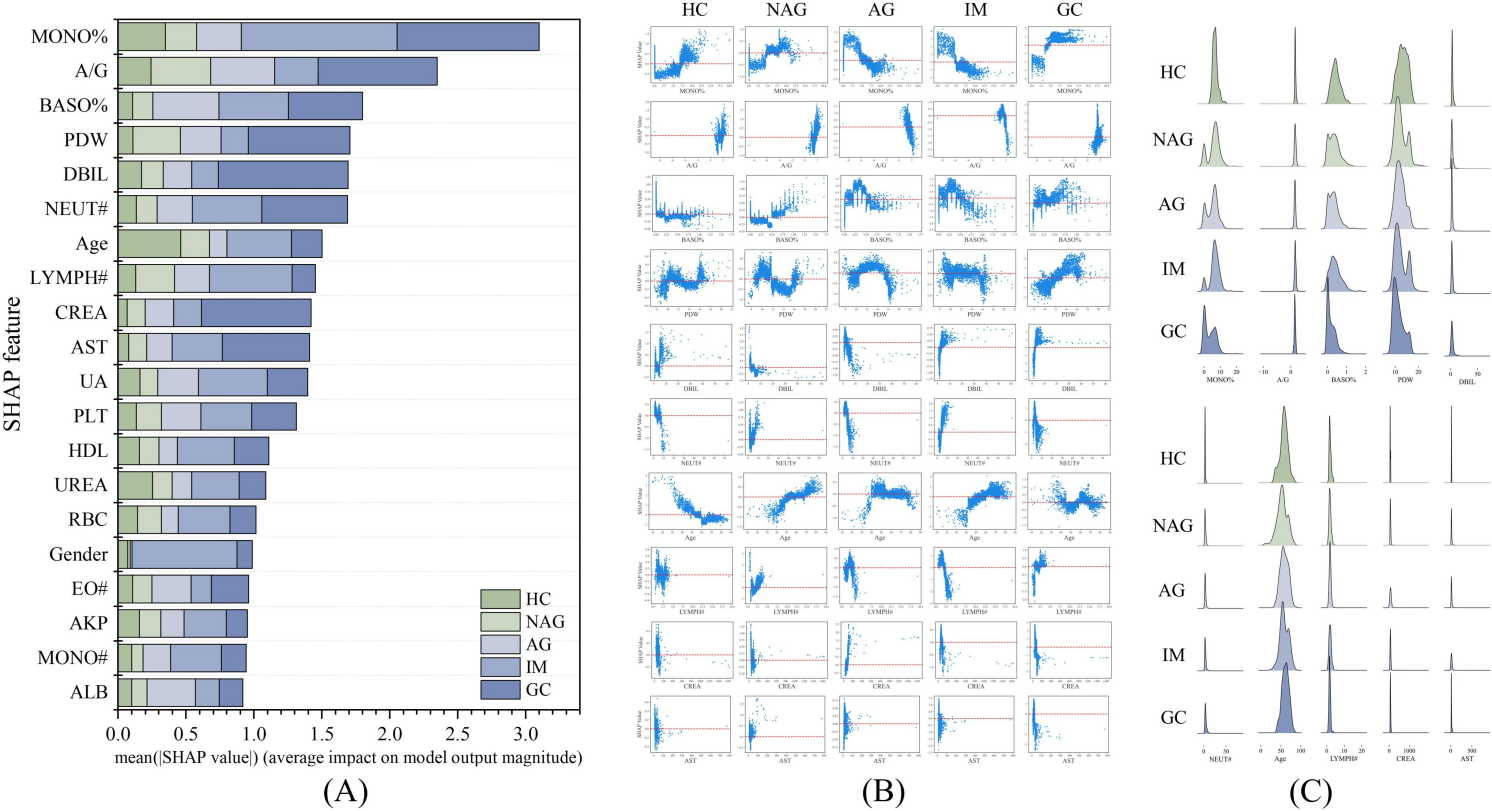
Global model interpretation using the SHAP method. (A) SHAP summary bar plot. (B) SHAP dependence plots for the top 10 features. Each dependence plot shows how a single feature affects the output of the prediction model, and each dot represents a single patient. (C) Density distributions of the top 10 features at different stages. A/G: albumin/globulin ratio; AG: atrophic gastritis; AKP: alkaline phosphatase; ALB: albumin; AST: aspartate aminotransferase; BASO%: basophil percentage; CREA: creatinine; DBIL: total bilirubin; EO#: eosinophil count; GC: gastric cancer; HC: healthy control; IM: intestinal metaplasia; LYMPH#: lymphocyte count; MONO#: monocyte count; MONO%: monocyte percentage; NAG: nonatrophic gastritis; NEUT#: neutrophil count; PDW: platelet distribution width; PLT: platelet count; SHAP: Shapley Additive Explanations; UA: uric acid; UREA: urea.

SHAP explainability analysis is used to gain further insight into the model’s decision-making process. Figure 4A presents the top 20 most important features in the model’s decision-making process, quantifying the significance of these features at different stages. For instance, MONO% plays a crucial role at the IM and GC stages, while DBIL is particularly important at the GC stage. Figure 4B shows how the top 10 features influence the decision boundary of the predicted outcomes. The results reveal that different features exhibit distinct threshold effects across different disease stages. For example, the threshold range for MONO% is between 3 and 7.5, which closely aligns with its clinical reference range (3-10), while the threshold range for DBIL is between 2 and 6, also close to its clinical reference range (0‐6.8). These findings suggest that diagnostic models built on these indicators have the potential to assist in disease screening and staging. The density distribution plot in Figure 4C further illustrates the distribution of the top 10 features across different stages. For example, indicators such as MONO%, BASO%, and NEUT# show significant differences in distribution and intensity between the HC and GC groups, confirming that these features play a crucial role in distinguishing between different disease stages. Additionally, since age is among the top 10 most important features, we further evaluated the model’s performance across different age strata in 2 external validation cohorts. The results showed that the CatBoost model continued to effectively differentiate patient data across various age strata. Detailed results are provided in Multimedia Appendix 7.

### Web Server Development

**Figure 5.**
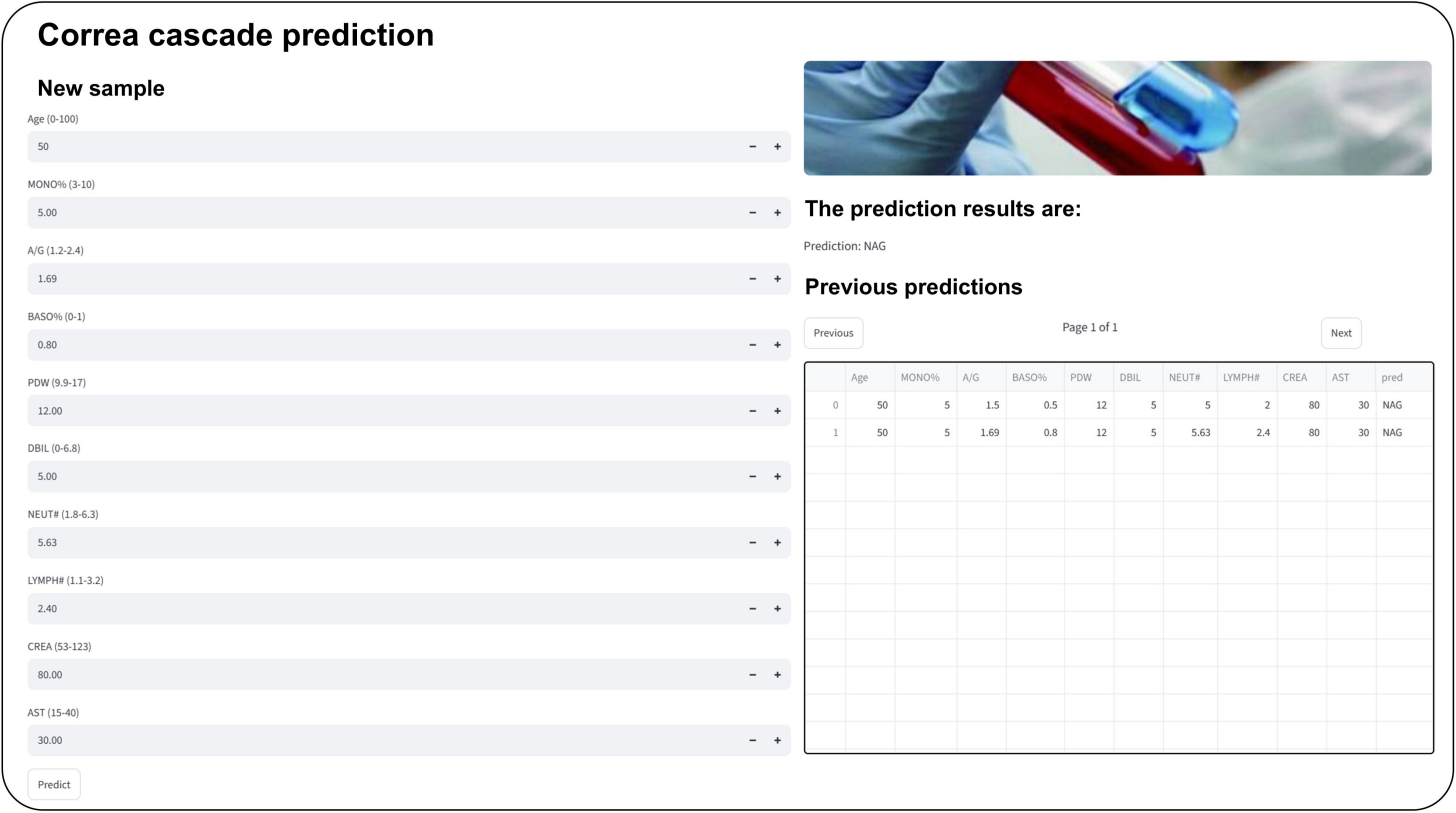
Web page tool presentation of the optimal CatBoost model. The final model includes 10 features for predicting different stages. After the 10 features are entered, the prediction result will be automatically displayed on the right side. A/G: albumin/globulin ratio; AST: aspartate aminotransferase; BASO: basophils; CatBoost: categorical boosting; CREA: creatinine; DBIL: total bilirubin; LYMPH#: lymphocyte count; NAG: nonatrophic gastritis; NEUT#: neutrophil count; PDW: platelet distribution width; UA: uric acid.

To maximize efficient use of the model and enhance its translational potential, we embedded a model constructed from the top 10 features ranked by SHAP into the Streamlit platform (Figure 5). Users need to enter only the 10 required features, and the application will automatically predict the stage of gastric disease progression for a given patient. This web tool is freely accessible through direct access to the Streamlit platform [27].

## Discussion

### Principal Findings

This study developed and validated an explainable ML-based model for predicting different stages in the progression of *H pylori*–initiated IGC. The model demonstrated robust performance across internal validation and external cohorts, suggesting that the ML-based model may serve as a potential tool for IGC staging. It supports early screening and facilitates informed clinical decision-making for subsequent evaluation and treatment.

### Comparison With Prior Work

Currently, most diagnostic models for gastric diseases based on routine laboratory indicators focus on binary classification at a single disease stage. Previous studies have primarily focused on discriminating between IM and NAG [28], the prediction of future GC risk in initially negative individuals [29], the assessment of laboratory parameter changes before and after treatment in patients with GC [30], or differentiation between GC and precancerous lesions [31]. In contrast, dynamic changes in hematological parameters across different stages of *H pylori–*initiated IGC remain poorly understood, as well as the feasibility of using these parameters for multistage prediction models also remains unclear.

In this study, we constructed a multiclass ML model for predicting different developmental stages of IGC. Among the evaluated models, CatBoost achieved the best diagnostic performance, with an accuracy of 0.8091 in the internal validation cohort and accuracies of 0.7996 and 0.8337 in 2 independent test sets, respectively. DCA further supported its potential clinical applicability. However, the relatively large number of features included in the current model may limit its practical implementation. Therefore, it will be important to develop more lightweight models and identify a more concise set of key features while maintaining predictive performance.

Given the lack of unified guidelines for feature selection in predictive models, SHAP analysis was used to provide both global and local explanations of the model, thereby elucidating the contribution of individual features to the model’s predictions [32]. This study demonstrates that routine laboratory indicators, including MONO%, A/G, BASO%, PDW, and DBIL, play important roles in distinguishing different disease stages. Higher MONO% was associated with an increased predicted risk of GC, which is consistent with previous findings showing that elevated monocyte proportion is associated with poorer prognosis and reduced overall survival [33]. In contrast, lower A/G levels markedly increased the likelihood of GC classification, in agreement with epidemiological evidence linking low A/G levels to increased mortality across multiple malignancies, including GC [34]. In addition, age was ranked among the top 10 most important features. Given that the incidence of GC increases significantly with age [35], we further evaluated the model’s predictive performance across different age strata. The results demonstrated that the CatBoost model was able to consistently identify patients at different disease stages across age groups. Furthermore, based on the top 10 key features and the Streamlit framework, we developed a user-friendly online prediction platform to enhance its clinical accessibility.

### Study Limitations

This study has the following limitations. First, it was based on populations at 2 hospitals in China, which may have been influenced by factors such as demographic structure and lifestyle habits. Therefore, the generalizability of the results needs further validation. However, the study provides preliminary evidence for diagnosing IGC based on routine laboratory hematological indicators. Second, although the model can distinguish between different stages of IGC progression to some extent, its relatively high specificity and slightly lower sensitivity may lead to missed or incorrect classifications, especially in late-stage patients, which is unacceptable for practical applications. Therefore, the current method should be used only as an auxiliary diagnostic tool and should be combined with routine clinical methods. Additionally, the construction of ML models requires sufficiently large, balanced, and representative clinical datasets. This study exhibits biases in the HC, AG, and GC groups. Although clinical data augmentation using the SMOTE method has been widely applied [25,26], its assumption of a uniform feature space means the synthetic data generated may not fully reflect real clinical heterogeneity or maintain complete clinical interpretability, potentially increasing the risk of overfitting. The relatively small external validation cohorts may also be insufficient to comprehensively assess the model’s generalizability. Therefore, future research should expand the sample size and include data from more collaborative centers to enhance the model’s generalizability. Moreover, the testing platforms differ across hospitals and may affect routine laboratory indicators and thus may influence model transferability. Although the study did not demonstrate the specific effects of platform differences on diagnostic outcomes in the results section, interinstrument and interlaboratory variability remains an important practical barrier to broader application. Therefore, promoting mutual recognition of test results and standardization of clinical reference ranges at regional and national levels is crucial. Finally, this study focuses only on IGC caused by *H pylori* infection, and its applicability to other types of GC, such as diffuse GC or signet-ring cell carcinoma, has not been clarified. Future studies should expand the scope of research to evaluate the prospects of using routine laboratory indicators in different types of GC.

### Conclusions

In conclusion, this study developed and validated an interpretable ML model based on routine clinical laboratory data to predict different stages in the progression of *H pylori* infection-associated IGC. The model demonstrated promising predictive performance in the internal validation and provided preliminary evidence of transportability in 2 external cohorts. As a potential auxiliary diagnostic tool, it shows promising applicability in remote areas and health care settings with limited medical resources, helping to optimize resource allocation and support early clinical intervention.

## Supplementary material

10.2196/94837 Multimedia Appendix 1 Grid search ranges and optimal parameter combinations for different machine learning algorithms.

10.2196/94837 Multimedia Appendix 2 Baseline characteristics of the Guangdong Provincial People’s Hospital (GDPH) and Shengli Oilfield Central Hospital (SOCH) cohort training sets after synthetic minority oversampling technique (SMOTE) augmentation.

10.2196/94837 Multimedia Appendix 3 Flowchart of the study design.

10.2196/94837 Multimedia Appendix 4 Baseline characteristics of the external validation in Guangdong Provincial People’s Hospital (GDPH) cohorts.

10.2196/94837 Multimedia Appendix 5 Baseline characteristics of the external validation in Shengli Oilfield Central Hospital (SOCH) cohorts.

10.2196/94837 Multimedia Appendix 6 Comparison of model performance across different correlation coefficients.

10.2196/94837 Multimedia Appendix 7 Age-stratified diagnostic performance of the model in the external validation cohort.

## Abbreviations

A/G: albumin/globulin ratio
AdaBoost: adaptive boosting
AG: atrophic gastritis
AKP: alkaline phosphatase
ALB: albumin
AST: aspartate aminotransferase
AUC: area under the curve
BASO%: basophil percentage
CatBoost: categorical boosting
CREA: creatinine
CRP: C-reactive protein
CV: cross-validation
DBIL: total bilirubin
DCA: decision curve analysis
DT: decision tree
EO#: eosinophil count
GC: gastric cancer
GDPH: Guangdong Provincial People’s Hospital
GGT: gamma-glutamyl transferase
GLU: glucose
HC: healthy control
HDL: high-density lipoprotein cholesterol
IARC: International Agency for Research on Cancer
IGC: intestinal-type gastric cancer
IL-6: interleukin-6
IM: intestinal metaplasia
KNN: k-nearest neighbor
LGBM: light gradient boosting machine
LIS: laboratory information systems
LYMPH#: lymphocyte count
MCH: mean corpuscular hemoglobin
MCHC: mean corpuscular hemoglobin concentration
ML: machine learning
MONO%: monocyte percentage
MPV: mean platelet volume
NAG: nonatrophic gastritis
NCC: National Cancer Center of China
NEUT#: neutrophil count
NLR: neutrophil-to-lymphocyte ratio
PCT: procalcitonin
PDW: platelet distribution width
PLR: platelet-to-lymphocyte ratio
RBC: red blood cell
RDW: red cell distribution width
RF: random forest
ROC: receiver operating characteristic
SHAP: Shapley Additive Explanations
SMOTE: synthetic minority oversampling technique
SOCH: Shengli Oilfield Central Hospital
TC: total cholesterol
TG: triglycerides
UA: uric acid
UREA: urea
WBC: white blood cell
XGBoost: extreme gradient boosting
