# Supplementary material for: Development and Validation of an Interpretable Machine Learning Model for Staging Helicobacter pylori–Initiated Intestinal-Type Gastric Cancer in the Correa Cascade: Cross-Sectional Study
Source: J Med Internet Res. 2026 Aug 7;28:e94837. doi: 10.2196/94837 (PMC13451052; doi:10.2196/94837)
Supplement: Supplementary file 1 [file jmir-v28-e94837-s001.docx]

**Multimedia Appendix 6** Gridsearch ranges and optimal parameter combinations for different machine learning algorithms.

| **Algorithms** | **Parameter Range** | **Optimum Parameter** |
| --- | --- | --- |
| **AdaBoost** | **learning_rate** = [0.1, 1, 0.01, 0.001],  **n_estimators** = [3, 5, 7, 10, 15, 20, 50, 60, 80, 100, 120, 130, 140, 145, 150, 160, 170, 175, 180, 185] | **learning_rate** = 1,  **n_estimators** = 160 |
| **CatBoost** | **learning_rate** = [0.1, 1, 0.01, 0.001, 0.0001],  **max_depth** = [3, 4, 5, 6, 7, 8, 9, 10, 11, 12, 15, 20, 25, 30] | **max_depth** = 10,  **learning_rate** = 0.01 |
| **Decision Tree** | **criterion** = ['gini', 'entropy'],  **max_depth** = range (1, 30), **max_features** = [3, 5, 7, 10, 15, 20, 21, 22, 23, 24, 25, 26, 28, 29, 30, 31, 32, 33, 34, 35, 36, 38, 39, 40] | **criterion** = 'gini',  **max_depth** = 16,  **max_features** = 22 |
| **LGBM** | **n_estimators =** [3, 5, 7, 10, 15, 20, 50, 60, 80, 100, 120, 130, 140, 145, 150, 160, 165, 170, 175, 180, 185],  **learning_r =** [0.1, 1, 0.01, 0.001] | **learning_rate** = 0.1,  **n_estimators** = 165 |
| **Random Forest** | **Criterion** = ['gini', 'entropy'],  **max_depth** = range (1, 20),  **n_estimators** = [3, 5, 7, 10, 15, 20, 50, 60, 80, 100, 120, 130, 140, 145, 150, 160, 170, 175, 180, 185] | **criterion** = 'entropy',  **max_depth** = 14, **n_estimators** = 140 |
| **XGBoost** | **n_estimators =** [50, 60, 70, 80, 90, 100, 110, 120, 130, 140, 150, 160, 170, 180],  **learning_r =** [0.1, 1, 0.01, 0.001] | **learning_rate** = 0.1,  **n_estimators** = 80 |

Abbreviations: AdaBoost: Adaptive Boosting; CatBoost: Categorical Boosting; DT: Decision Tree algorithm; LGBM: Light Gradient Boosting Machine; RF: Random Forest; XGBoost: eXtreme Gradient Boosting.
