# Supplementary material for: Development and Validation of an Interpretable Machine Learning Model for Staging Helicobacter pylori–Initiated Intestinal-Type Gastric Cancer in the Correa Cascade: Cross-Sectional Study
Source: J Med Internet Res. 2026 Aug 7;28:e94837. doi: 10.2196/94837 (PMC13451052; doi:10.2196/94837)
Supplement: Supplementary file 2 [file jmir-v28-e94837-s002.docx]

**Multimedia Appendix 1** Baseline characteristics of the GDPH and SOCH cohort training sets after SMOTE augmentation.

| **Characteristics** | **Training set (n=3639)** | | | | | **P** |
| --- | --- | --- | --- | --- | --- | --- |
|  | HC (n=780) | NAG (n=703) | AG (n=780) | IM (n=586) | GC (n=790) |  |
| Male, n/total (%) | 413/780 (52.90) | 421/703 (59.80) | 545/780 (69.80) | 387/586 (66.00) | 606/790 (76.70) | <0.001 |
| Age (years) | 59.00 (53.00-64.00) | 53.00 (47.00-61.00) | 58.50 (52.00-66.00) | 58.00 (52.00-68.00) | 62.00 (56.00-68.00) | <0.001 |
| AST | 20.80 (18.80-23.30) | 20.00 (17.00-24.00) | 20.60 (17.70-24.58) | 21.00 (17.00-25.00) | 19.00 (16.68-22.30) | <0.001 |
| ALB | 42.91 (40.54-45.35) | 41.80 (39.20-44.60) | 40.81 (38.50-43.18) | 41.90 (39.48-44.20) | 40.79 (38.00-43.40) | <0.001 |
| A/G | 1.60 (1.50-1.74) | 1.60 (1.40-1.80) | 1.50 (1.32-1.66) | 1.60 (1.50-1.80) | 1.46 (1.33-1.61) | <0.001 |
| DBIL | 3.10 (2.60-3.89) | 2.50 (1.90-3.30) | 2.40 (1.91-3.10) | 2.50 (2.00-3.50) | 2.47 (1.85-3.58) | <0.001 |
| AKP | 73.53 (63.54-84.09) | 69.00 (56.00-84.00) | 71.69 (62.04-85.52) | 70.10 (59..00-83.25) | 72.89 (60.28-88.51) | <0.001 |
| GGT | 20.98 (15.77-26.99) | 24.00 (16.00-39.00) | 23.99 (15.79-37.33) | 22.00 (16.00-37.00) | 19.00 (15.68-25.76) | <0.001 |
| GLU | 5.22 (4.82-5.87) | 5.11 (4.61-5.79) | 5.19 (4.77-5.90) | 5.09 (4.61-5.88) | 5.36 (4.78-6.07) | <0.001 |
| UREA | 5.26 (4.33-6.21) | 4.95 (4.27-5.82) | 5.18 (4.48-6.27) | 5.22 (4.45-6.30) | 5.45 (4.65-6.20) | <0.001 |
| CREA | 55.67 (49.43-66.02) | 64.70 (54.40-76.90) | 66.91 (58.27-83.08) | 66.80 (55.60-76.90) | 66.40 (57.24-75.61) | <0.001 |
| UA | 295.01 (237.69-362.11) | 337.40 (268.00-404.00) | 350.69 (289.91-415.49) | 328.95 (270.98-392.25) | 301.76 (246.17-362.61) | <0.001 |
| TC | 4.90 (4.34-5.51) | 4.96 (4.16-5.69) | 5.14 (4.51-5.71) | 4.87 (4.20-5.61) | 4.70 (3.98-5.27) | <0.001 |
| TG | 1.21 (1.02-1.50) | 1.32 (0.98-1.94) | 1.41 (1.03-2.10) | 1.35 (0.98-1.93) | 1.32 (1.03-1.74) | <0.001 |
| HDL | 1.41 (1.15-1.64) | 1.19 (1.00-1.46) | 1.23 (1.06-1.43) | 1.20 (1.02-1.48) | 1.25 ± 0.24 | <0.001 |
| MONO% | 6.75 (5.75-7.56) | 6.80 (5.00-8.10) | 6.00 (2.53-7.35) | 6.70 (5.50-8.30) | 3.15 (0.09-6.48) | <0.001 |
| BA% | 0.41 (0.28-0.59) | 0.30 (0.10-0.50) | 0.30 (0.11-0.46) | 0.40 (0.20-0.60) | 0.13 (0.01-0.33) | <0.001 |
| NE | 3.06 (2.72-3.45) | 3.25 (2.63-4.35) | 3.38 (2.75-4.02) | 3.46 (2.68-4.41) | 4.12 (3.27-5.31) | <0.001 |
| LY | 1.85 (1.54-2.30) | 1.83 (1.47-2.25) | 1.90 (1.63-2.23) | 1.91 (1.46-2.36) | 1.66 (1.40-2.00) | <0.001 |
| MO | 0.36 (0.31-0.45) | 0.42 (0.33-0.52) | 0.44 (0.35-0.55) | 0.42 (0.35-0.56) | 0.48 (0.38-0.56) | <0.001 |
| EO | 0.10 (0.07-0.14) | 0.12 (0.07-0.20) | 0.15 (0.07-0.20) | 0.11 (0.06-0.20) | 0.11 (0.07-0.17) | <0.001 |
| RBC | 4.43 (4.20-4.77) | 4.56 (4.20-4.90) | 4.51 (4.18-4.81) | 4.59 (4.24-4.90) | 4.34 (3.99-4.61) | <0.001 |
| MCH | 30.20 (29.50-31.40) | 30.10 (29.00-31.40) | 30.60 (29.53-31.68) | 30.70 (29.60-31.70) | 30.50 (29.38-31.70) | <0.001 |
| MCHC | 335.00 (330.00-342.00) | 335.00 (327.00-342.00) | 336.00 (327.00-342.00) | 337.00 (330.00-344.00) | 333.00 (327.00-340.00) | <0.001 |
| PLT | 223.50 (200.00-261.75) | 229.00 (194.00-277.00) | 219.00 (184.00-266.00) | 218.50 (182.00-263.00) | 238.00 (204.00-304.25) | <0.001 |
| MPV | 10.21 (9.39-10.88) | 10.10 (9.40-10.60) | 10.10 (9.42-10.67) | 9.90 (9.30-10.60) | 9.70 (9.09-10.21) | <0.001 |
| PDW | 13.27 (11.61-14.99) | 11.90 (10.50-13.90) | 12.00 (10.60-13.58) | 11.70 (10.40-14.40) | 10.92 (9.64-12.96) | <0.001 |

^*^NOTE: Data are presented as numbers (%), mean ± SD, and median [interquartile range]. AST aspartate aminotransferase, ALB albumin, A/G albumin/globulin ratio, DBIL total bilirubin, AKP alkaline phosphatase, GGT gamma-glutamyl transferase, GLU glucose, UREA urea, CREA creatinine, UA uric acid, TC total cholesterol, TG triglycerides, HDL high-density lipoprotein cholesterol, MONO% monocyte percentage, BASO% basophil percentage, NEUT# neutrophil count, LYMPH# lymphocyte count, MONO# monocyte count, EO# eosinophil count, RBC red blood cell count, MCH mean corpuscular hemoglobin, MCHC mean corpuscular hemoglobin concentration, PLT platelet count, MPV mean platelet volume, PDW platelet distribution width. *P value*: HC vs NAG vs AG vs IM vs GC.
