# Supplementary material for: Development and Validation of an Interpretable Machine Learning Model for Staging Helicobacter pylori–Initiated Intestinal-Type Gastric Cancer in the Correa Cascade: Cross-Sectional Study
Source: J Med Internet Res. 2026 Aug 7;28:e94837. doi: 10.2196/94837 (PMC13451052; doi:10.2196/94837)
Supplement: Supplementary file 3 [file jmir-v28-e94837-s003.docx]

**Multimedia Appendix 3** Flow chart of the study design. HC: Healthy Control; NAG: Non-Atrophic Gastritis; AG: Atrophic Gastritis; IM: Intestinal Metaplasia; GC: Gastric Cancer; GDPH: Guangdong Provincial People’s Hospital; SOCH: Shengli Oilfield Central Hospital; ROC: Receiver Operating Characteristic; SHAP: SHapley Additive exPlanations; KNN: K-Nearest Neighbors; SMOTE: Synthetic Minority Over-sampling Technique; CV: Cross-Validation; LightGBM: Light Gradient Boosting Machine; XGBoost: Extreme Gradient Boosting.
