# Supplementary material for: Development and Validation of an Interpretable Machine Learning Model for Staging Helicobacter pylori–Initiated Intestinal-Type Gastric Cancer in the Correa Cascade: Cross-Sectional Study
Source: J Med Internet Res. 2026 Aug 7;28:e94837. doi: 10.2196/94837 (PMC13451052; doi:10.2196/94837)
Supplement: Supplementary file 4 [file jmir-v28-e94837-s004.docx]

**Multimedia Appendix 4** Baseline characteristics of the external validation in GDPH cohorts.

| **Characteristics**  **GDPH** | **External validation cohort (n=50)** | | | ***P value*** |
| --- | --- | --- | --- | --- |
|  | AG (n=30) | IM (n=10) | GC (n=20) |  |
| Male, n/total (%) | 21/30 (70.00) | 6/10 (60.00) | 9/10 (90.00) | 0.262 |
| Age (years) | 56.47 ± 10.56 | 55.90 ± 18.58 | 60.10 ± 11.35 | 0.673 |
| AST | 19.50 (16.00-26.25) | 21.50 (17.50-27.25) | 17.00 (13.25-24.50) | 0.299 |
| ALB | 41.25 ± 3.80 | 43.07 ± 4.04 | 37.18 ± 3.53 | 0.003 |
| A/G | 1.34 ± 0.27 | 1.46 ± 0.38 | 1.40 ± 0.20 | 0.514 |
| DBIL | 2.30 (1.50-3.48) | 3.19 ± 1.29 | 1.95 (1.50-2.90) | 0.173 |
| AKP | 71.07 ± 18.27 | 85.00 (60.50-98.00) | 60.60 ± 25.78 | 0.213 |
| GGT | 29.50 (19.75-39.00) | 25.00 (16.25-30.00) | 25.50 (12.25-45.00) | 0.354 |
| GLU | 5.04 (4.65-5.47) | 5.04 (4.71-5.57) | 4.45 (3.52-4.96) | 0.079 |
| UREA | 5.36 ± 1.43 | 4.48 ± 1.25 | 6.20 ± 1.99 | 0.051 |
| CREA | 79.22 ± 19.11 | 66.17 ± 14.76 | 89.03 ± 24.29 | 0.039 |
| UA | 395.35 ± 88.84 | 292.22 ± 65.92 | 340.70 ± 130.05 | 0.012 |
| TC | 5.60 ± 1.53 | 4.88 ± 1.69 | 5.01 ± 1.31 | 0.339 |
| TG | 1.33 (0.99-2.20) | 1.06 ± 0.30 | 1.33 (1.06-1.51) | 0.163 |
| HDL | 1.32 ± 0.37 | 1.26 ± 0.45 | 0.97 ± 0.23 | 0.041 |
| MONO% | 0.08 ± 0.02 | 0.08 ± 0.01 | 0.08 ± 0.01 | 0.757 |
| BA% | 0.004 (0.003-0.006) | 0.003 ± 0.001 | 0.005 ± 0.003 | 0.7 |
| NE | 3.71 ± 1.26 | 3.68 ± 1.17 | 5.34 ± 1.37 | 0.003 |
| LY | 2.06 ± 0.53 | 2.16 ± 0.77 | 1.52 ± 0.59 | 0.034 |
| MO | 0.51 ± 0.16 | 0.48 ± 0.08 | 0.59 ± 0.14 | 0.261 |
| EO | 0.18 (0.09-0.32) | 0.09 (0.08-0.18) | 0.17 (0.04-0.40) | 0.429 |
| RBC | 4.70 ± 0.63 | 4.51 ± 0.60 | 4.17 ± 0.59 | 0.068 |
| MCH | 30.00 (28.28-31.08) | 30.95 (26.60-31.45) | 28.72 ± 3.22 | 0.804 |
| MCHC | 328.70 ± 12.32 | 330.00 (326.50-343.50) | 327.50 ± 12.88 | 0.823 |
| PLT | 255.50 (201.50-280.00) | 284.60 ± 70.93 | 324.70 ± 135.36 | 0.14 |
| MPV | 9.67 ± 1.12 | 9.53 ± 0.83 | 9.40 (9.23-10.45) | 0.954 |
| PDW | 10.73 ± 2.16 | 10.21 ± 1.68 | 9.55 (8.70-11.73) | 0.789 |

^*^NOTE: Data are presented as numbers (%), mean ± SD, and median [interquartile range]. AST aspartate aminotransferase, ALB albumin, A/G albumin/globulin ratio, DBIL total bilirubin, AKP alkaline phosphatase, GGT gamma-glutamyl transferase, GLU glucose, UREA urea, CREA creatinine, UA uric acid, TC total cholesterol, TG triglycerides, HDL high-density lipoprotein cholesterol, MONO% monocyte percentage, BASO% basophil percentage, NEUT# neutrophil count, LYMPH# lymphocyte count, MONO# monocyte count, EO# eosinophil count, RBC red blood cell count, MCH mean corpuscular hemoglobin, MCHC mean corpuscular hemoglobin concentration, PLT platelet count, MPV mean platelet volume, PDW platelet distribution width. *P value*: HC vs NAG vs AG vs IM vs GC.
