# Supplementary material for: Development and Validation of an Interpretable Machine Learning Model for Staging Helicobacter pylori–Initiated Intestinal-Type Gastric Cancer in the Correa Cascade: Cross-Sectional Study
Source: J Med Internet Res. 2026 Aug 7;28:e94837. doi: 10.2196/94837 (PMC13451052; doi:10.2196/94837)
Supplement: Supplementary file 5 [file jmir-v28-e94837-s005.docx]

**Multimedia Appendix 2** Baseline characteristics of the external validation in SOCH cohorts.

| **Characteristics**  **SOCH** | **External validation cohort (n=90)** | | | | | ***P value*** |
| --- | --- | --- | --- | --- | --- | --- |
|  | HC (n=20) | NAG (n=20) | AG (n=20) | IM (n=20) | GC (n=10) |  |
| Male, n/total (%) | 7/20 (35.00) | 10/20 (50.00) | 11/20 (55.00) | 17/20 (85.00) | 6/10 (n=60.00) | 0.019 |
| Age (years) | 52.55 ± 11.84 | 51.85 ± 7.41 | 52.00 (50.25-54.00) | 60.20 ± 10.55 | 68.50 (62.25-72.50) | 0.001 |
| AST | 21.30 ± 4.90 | 19.85 ± 4.97 | 22.50 (16.50-28.00) | 20.00 (16.00-24.75) | 20.00 (17.75-42.25) | 0.671 |
| ALB | 43.63 ± 3.53 | 42.76 ± 4.36 | 41.84 ± 4.09 | 43.09 ± 3.65 | 40.87 ± 6.67 | 0.565 |
| A/G | 1.55 ± 0.17 | 43.85 (39.75-46.03) | 1.62 ± 0.32 | 1.59 ± 0.21 | 1.56 ± 0.34 | 0.607 |
| DBIL | 2.90 ± 1.22 | 2.45 (2.00-4.38) | 2.35 (1.93-3.60) | 2.50 (2.05-3.05) | 2.85 ± 0.76 | 0.982 |
| AKP | 74.00 ± 19.99 | 76.55 ± 20.79 | 75.40 ± 29.27 | 76.25 ± 22.44 | 77.70 ± 20.51 | 0.994 |
| GGT | 21.00 (15.50-39.75) | 21.00 (13.25-32.25) | 24.50 (15.00-44.50) | 24.00 (18.00-66.00) | 17.50 (15.00-25.25) | 0.284 |
| GLU | 5.20 (4.66-5.86) | 5.19 (4.91-5.54) | 5.39 (4.85-5.84) | 5.47 (4.84-6.28) | 6.05 ± 1.44 | 0.67 |
| UREA | 4.78 ± 1.62 | 5.39 ± 1.46 | 5.47 (4.24-7.47) | 5.21 ± 1.34 | 5.63 ± 1.93 | 0.331 |
| CREA | 54.90 (50.50-64.48) | 63.24 ± 14.41 | 64.80 (52.00-70.43) | 67.90 ± 14.35 | 59.49 ± 12.92 | 0.241 |
| UA | 340.90 ± 93.51 | 314.07 ± 103.90 | 347.99 ± 99.33 | 388.51 ± 132.69 | 337.59 ± 117.92 | 0.314 |
| TC | 5.18 ± 1.00 | 4.54 ± 0.92 | 4.95 ± 1.13 | 5.26 ± 1.26 | 4.90 ± 1.16 | 0.274 |
| TG | 1.22 (1.04-1.89) | 1.38 (0.86-2.43) | 1.37 (0.81-3.46) | 1.79 (1.22-2.23) | 1.15 ± 0.34 | 0.206 |
| HDL | 1.32 (1.03-1.89) | 1.21 (0.97-1.29) | 1.21 ± 0.25 | 1.25 (1.01-1.38) | 1.37 ± 0.35 | 0.365 |
| MONO% | 7.17 ± 2.86 | 6.30 (4.85-8.25) | 7.25 ± 2.44 | 6.52 ± 1.39 | 6.89 ± 2.20 | 0.94 |
| BA% | 0.30 ± 0.25 | 0.45 ± 0.28 | 0.48 ± 0.26 | 0.40 (0.40-0.58) | 0.20 (0.18-0.38) | 0.05 |
| NE | 3.18 (2.73-4.17) | 3.42 (2.30-4.28) | 3.01 (2.02-3.62) | 2.91 (2.58-3.64) | 3.90 (2.63-5.77) | 0.425 |
| LY | 1.86 ± 0.57 | 1.94 ± 0.38 | 1.79 ± 0.48 | 1.81 ± 0.50 | 1.69 ± 0.59 | 0.742 |
| MO | 0.34 (0.27-0.59) | 0.34 (0.27-0.56) | 0.39 ± 0.11 | 0.35 ± 0.08 | 0.45 ± 0.18 | 0.845 |
| EO | 0.07 (0.04-0.12) | 0.09 (0.07-0.12) | 0.09 (0.05-0.19) | 0.12 (0.06-0.17) | 0.07 (0.04-0.16) | 0.464 |
| RBC | 4.69 ± 0.55 | 4.40 (4.14-4.99) | 4.60 ± 0.49 | 4.57 ± 0.59 | 4.17 ± 0.66 | 0.197 |
| MCH | 29.75 (29.15-31.35) | 29.63 ± 2.03 | 30.45 (29.20-31.85) | 31.10 (29.88-32.53) | 30.97 ± 1.56 | 0.157 |
| MCHC | 330.80 ± 9.88 | 335.2 ± 11.56 | 336.75 ± 15.57 | 334.65 ± 15.23 | 334.20 ± 7.47 | 0.673 |
| PLT | 233.45 ± 70.12 | 227.20 ± 34.79 | 225.60 ± 77.60 | 207.25 ± 56.88 | 214.70 ± 68.73 | 0.682 |
| MPV | 10.03 ± 0.89 | 10.01 ± 1.01 | 10.30 ± 0.98 | 9.73 ± 1.19 | 9.62 ± 1.02 | 0.359 |
| PDW | 15.30 (12.10-16.15) | 14.55 (12.08-16.23) | 13.06 ± 2.34 | 15.70 (11.58-16.38) | 15.80 (11.88-16.23) | 0.507 |

^*^NOTE: Data are presented as numbers (%), mean ± SD, and median [interquartile range]. AST aspartate aminotransferase, ALB albumin, A/G albumin/globulin ratio, DBIL total bilirubin, AKP alkaline phosphatase, GGT gamma-glutamyl transferase, GLU glucose, UREA urea, CREA creatinine, UA uric acid, TC total cholesterol, TG triglycerides, HDL high-density lipoprotein cholesterol, MONO% monocyte percentage, BASO% basophil percentage, NEUT# neutrophil count, LYMPH# lymphocyte count, MONO# monocyte count, EO# eosinophil count, RBC red blood cell count, MCH mean corpuscular hemoglobin, MCHC mean corpuscular hemoglobin concentration, PLT platelet count, MPV mean platelet volume, PDW platelet distribution width. *P value*: HC vs NAG vs AG vs IM vs GC.
