# Supplementary material for: Development and Validation of an Interpretable Machine Learning Model for Staging Helicobacter pylori–Initiated Intestinal-Type Gastric Cancer in the Correa Cascade: Cross-Sectional Study
Source: J Med Internet Res. 2026 Aug 7;28:e94837. doi: 10.2196/94837 (PMC13451052; doi:10.2196/94837)
Supplement: Supplementary file 6 [file jmir-v28-e94837-s006.docx]

**Multimedia Appendix 6** Comparison of model performance across different correlation coefficient.

| **correlation** | **Accuracy** | **Precision** | **Sensitivity** | **Specificity** | **F1** |
| --- | --- | --- | --- | --- | --- |
| 0.6 | 77.81% | 77.81% | 78.03% | 95.71% | 77.30% |
| 0.65 | 81.32% | 81.32% | 81.48% | 96.57% | 81.06% |
| 0.7 | 76.07% | 76.07% | 75.76% | 95.28% | 75.65% |
| 0.75 | 78.86% | 78.86% | 79.13% | 95.97% | 78.57% |
| 0.8 | 78.64% | 78.64% | 78.23% | 95.93% | 78.35% |
| 0.85 | 79.11% | 79.11% | 78.76% | 96.04% | 78.87% |
| 0.9 | 80.05% | 80.05% | 79.71% | 96.28% | 79.89% |
| 0.95 | 78.18% | 78.18% | 77.79% | 95.80% | 77.88% |
| 1 | 80.26% | 80.26% | 80.39% | 96.31% | 79.97% |
