# Supplementary material for: Development and Validation of an Interpretable Machine Learning Model for Staging Helicobacter pylori–Initiated Intestinal-Type Gastric Cancer in the Correa Cascade: Cross-Sectional Study
Source: J Med Internet Res. 2026 Aug 7;28:e94837. doi: 10.2196/94837 (PMC13451052; doi:10.2196/94837)
Supplement: Supplementary file 7 [file jmir-v28-e94837-s007.docx]

**Multimedia Appendix 5** Age-stratified diagnostic performance of the model in the external validation cohort.

| **Hospital** | **Age stratification** | **Accuracy** | **Precision** | **Sensitivity** | **Specificity** | **F1-score** |
| --- | --- | --- | --- | --- | --- | --- |
| **GDPH** | **28-44** | 0.7547 (0.6950-0.8000) | 0.7262 (0.6550-0.7900) | 0.7704 (0.7000-0.8350) | 0.7704 (0.7050-0.8350) | 0.7360 (0.6700-0.8000) |
|  | **45-59** | 0.8170 (0.6875-0.9167) | 0.8367 (0.7062-0.9489) | 0.8438 (0.7584-0.9222) | 0.8438 (0.7584-0.9222) | 0.8195 (0.7025-0.9197) |
|  | **≥60** | 0.8999 (0.8000-1.0000) | 0.8150 (0.6299-1.0000) | 0.8375 (0.7000-1.0000) | 0.8375 (0.7000-1.0000) | 0.8104 (0.6597-1.0000) |
| **SOCH** | **28-44** | 0.8752 (0.7250-1.0000) | 0.7658 (0.6000-1.0000) | 0.7658 (0.6000-1.0000) | 0.7658 (0.6000-1.0000) | 0.7658 (0.6000-1.0000) |
|  | **45-59** | 0.8297 (0.7957-0.9565) | 0.7858 (0.6000-0.8750) | 0.7175 (0.4374-0.9608) | 0.7175 (0.4374-0.9608) | 0.7403 (0.4687-0.8971) |
|  | **≥60** | 0.7313 (0.6263-0.8947) | 0.6856 (0.6042-0.8561) | 0.6687 (0.6037-0.8593) | 0.6687 (0.6037-0.8593) | 0.6673 (0.6079-0.8302) |
